# Supplementary material for: Development of an Event-Specific Droplet Digital PCR Assay for Quantification and Evaluation of the Transgene DNAs in Trace Samples of GM PRNP-Knockout Goat
Source: Foods. 2022 Mar 18;11(6):868. doi: 10.3390/foods11060868 (PMC8953510; doi:10.3390/foods11060868)
Supplement: Supplementary file 1 [file foods-11-00868-s001.zip › foods-1643314-supplementary.pdf]

Supplementary Material

Table S1. Information of practical samples.

Sample type	Sample name	Animal type	Goat generation
Milk	M1	GM KoP1 event	F3
	M2	GM KoP1 event	F4
	M3	GM KoP1 event	F3
	M4	GM KoP1 event	F5
Fresh Faces	F1	GM KoP1 event	F3
	F2	GM KoP1 event	F2
	F3	GM KoP1 event	F2
	F4	GM KoP1 event	F3
	F5	GM KoP1 event	F3
	F6	GM KoP1 event	F4
	F7	GM KoP1 event	F4
	F8	GM KoP1 event	F4
	F9	GM KoP1 event	F5
	F10	GM KoP1 event	F5
	F11	GM KoP1 event	F5
	F12	GM KoP1 event	F5
	F13	GM KoP1 event	F5
	F14	GM KoP1 event	F5
	F15	GM KoP1 event	F5
	F16	GM KoP1 event	F5
	F17	Non-GM	/
	F18	Non-GM	/
Compost Soil	S1	GM KoP1 event	/
	S2	GM KoP1 event	/
	S3	GM KoP1 event	/
	S4	GM KoP1 event	/
	S5	GM KoP1 event	/
